# Intratumoral Injection of Anlotinib Hydrogel Combined With Radiotherapy Reduces Hypoxia in Lewis Lung Carcinoma Xenografts: Assessment by Micro Fluorine-18-fluoromisonidazole Positron Emission Tomography/Computed Tomography Hypoxia Imaging

**DOI:** 10.3389/fonc.2021.628895

**Published:** 2021-03-12

**Authors:** Qin Gao, YiQing Jiang, XiaoJie Li, Hui Chen, Shan Tang, Han Chen, XiangXiang Shi, Yue Chen, ShaoZhi Fu, Sheng Lin

**Affiliations:** ^1^Department of Oncology, Affiliated Hospital of Southwest Medical University, Luzhou, China; ^2^Nuclear Medicine and Molecular Imaging Key Laboratory of Sichuan Province, Affiliated Hospital of Southwest Medical University, Luzhou, China

**Keywords:** Lewis lung carcinoma, hypoxia, anlotinib hydrogel, intratumoral injection, radiotherapy, micro fluorine-18-fluoromisonidazole positron emission tomography/computed tomography

## Abstract

Hypoxia is a common feature of solid tumors that increases tumor invasiveness and resistance to radiotherapy (RT) and chemotherapy. Local application of anlotinib (AL) might increase the regulation of new blood vessel growth and improve tumor hypoxia in RT. Therefore, it is essential to fully understand the drug delivery system of AL. Herein, we applied hypoxia imaging using micro fluorine-18-fluoromisonidazole positron emission tomography/computed tomography (micro 18F-FMISO PET/CT) to assess responses to intratumoral injections of an AL hydrogel (AL-HA-Tyr) combined with RT in mice bearing Lewis lung carcinoma (LLC). We formed AL-HA-Tyr by encapsulating AL with hyaluronic acid-tyramine (HA-Tyr) conjugates *via* the oxidative coupling of tyramine moieties catalyzed by H_2_O_2_ and horseradish peroxidase. AL-HA-Tyr restrained the proliferation of human umbilical endothelial cells (HUVECs) in colony formation assays *in vitro* (p < 0.001). We established a subcutaneous LLC xenograft model using C57BL/6J mice that were randomly assigned to six groups that were treated with AL, HA-Tyr, AL-HA-Tyr, RT, and RT+AL-HA-Tyr, or untreated (controls). Tumor volume and weight were dynamically measured. Post treatment changes in hypoxia were assessed in some mice using micro 18F-FMISO PET/CT, and survival was assessed in others. We histopathologically examined toxicity in visceral tissues and Ki-67, VEGF-A, *γ*-H2AX, and HIF-1*α* expression using immunohistochemistry. Direct intratumoral injections of AL-HA-Tyr exerted anti-tumor effects and improved hypoxia like orally administered AL (p > 0.05), but reduced visceral toxicity and prolonged survival. The uptake of 18F-FMISO did not significantly differ among the AL, AL-HA-Tyr, and RT+AL-HA-Tyr treated groups. Compared with the other agents, RT+AL-HA-Tyr decreased HIF-1*α*, Ki67, and VEGF-A expression, and increased *γ*-H2AX levels in tumor cells. Overall, compared with AL and AL-HA-Tyr, RT+AL-HA-Tyr improved tumor hypoxia, enhanced anti-tumor effects, and prolonged the survival of mice bearing LLC.

## Introduction

Lung cancer accounts for the highest morbidity worldwide. Approximately 75% patients are diagnosed in the middle and advanced stages, which seriously hampers treatment (1, 2). Hypoxia is a common feature of most solid tumors that increases tumor invasiveness and resistance to radiotherapy (RT) and chemotherapy, which is one important factor involved in poor prognoses (3). RT is the primary treatment option for patients with locally advanced (stage III) lung cancer (4).

Antiangiogenic drugs have the potential to make oxygen delivery more efficient and increase the efficacy of RT. Anlotinib (AL) hydrochloride is a new and promising antiangiogenesis drug, which has broad anti-tumor capacity showing significant therapeutic effect on several kinds of solid tumors (5–9). However, being an anti-vascular drug, many elderly patients cannot tolerate AL, due to side effects such as hypertension and hyperlipidemia. Therefore, we considered how to reduce side effects and maximize anti-tumor benefits. Hyaluronic acid is safe, non-toxic, and its derivatives have been widely applied as drug carriers (10). We previously loaded HA-Tyr with AL, synthesized AL-HA-Tyr, and verified its effectiveness and safety (11). Local application of AL might downregulate the angiogenesis and tumor hypoxia during RT. Therefore, the effects AL-HA-Tyr on angiogenesis, tumors, and hypoxia should be fully understood in the context of RT.

Most hypoxia studies have focused on relationships between the degree of hypoxia, the expression of vascular endothelial growth factor (VEGF), and rates of overall survival and local recurrence (12–14). The effects of intratumorally injected AL hydrogel (AL-HA-Tyr) on tumors and hypoxia when combined with RT have not yet been reported. Areas and degrees of tumor hypoxia can be directly assessed using convenient, minimally invasive, and reproducible, targeted molecular imaging using 18F-fluimidazole (18F-FMISO) PET/CT (15). This study established a local application platform and verified the effects of AL-HA-Tyr with radiotherapy therapy in Lewis lung carcinoma (LLC) tumor-bearing C57BL/6J mice using micro 18F-FMISO PET/CT.

## Materials and Methods

### Reagents and Cell Lines

Sodium hyaluronate (HA) (>95%, Mw = 90 kDa), tyramine hydrochloride (Tyr·HCl), 1-Ethyl-3-(3-dimethylaminopropyl)-carbodiimide hydrochloride (EDC·HCl), Hydrogen peroxide (H_2_O_2_, wt. 30%), N-hydroxysuccinimide (NHS), Horseradish peroxidase (HRP, 400–1,000 U/mg) and bovine testicular hyaluronidase were all purchased from MeiLun Co. Ltd. (Dalian, China). AL (dihydrochloride form; purity >99.9%) was obtained from Jiangsu Chia-tai Tianqing Pharmaceutical Co, Ltd (Nanjing, China). Crystal violet was purchased from Kelong Co. Ltd. (Chengdu, China). Polyclonal antibodies against Ki-67, VEGF-A, HIF-1*α*, and *γ*-H2AX were purchased from Bioworld Technology Co. Ltd. (Nanjing, China).

LLCs and human umbilical endothelial cells (HUVECs) were obtained from the experimental laboratory of Southwest Medical University (Luzhou, Sichuan, China) and cultured in a humidified 5% CO_2_ atmosphere at 37°C with Dulbecco’s Modified Eagle’s medium (HyClone; Thermo Scientific, Waltham, MA, USA), 10% fetal bovine serum (HyClone, Thermo Scientific), 0.1 mg/ml streptomycin and 100 U/ml penicillin.

### Synthesis of HA-Tyr Conjugates and AL-HA-Tyr

The HA-Tyr conjugates were prepared as follows (16): at 25°C, 2.5 mmol HA was dissolved in distilled water (100 ml), and 1.2 mmol tyramine hydrochloride was added. Then 2.5 mmol EDC·HCl and 2.5 mmol NHS were added to the solution to initiate the conjugation reaction at the constant pH of 4.7 with 0.1 M NaOH, and then the pH was adjusted to 7.0 overnight. The mixture was transferred to dialysis bags (molecular weight cutoff = 1,000 Da), and dialysis was carried out in 100 mM sodium chloride solution, a mixture of distilled water and ethanol (3:1) and distilled water for 1 day, separately. The HA-Tyr was obtained after the purified solution was lyophilized for 3 days and determined through proton nuclear magnetic resonance (^1^H-NMR). We prepared AL-HA–Tyr by dissolving HA–Tyr (1.0 wt.%) and AL (different concentrations) in distilled water, then HRP (50 U/ml) and H_2_O_2_ (20 mM) were added at 25°C.

### Determination of Amount of Time Required for HA-Tyr Hydrogelation

The gelation time of hydrogel was evaluated by continuously tilting the small tube (17) after HA-Tyr (1.0 wt.%), HRP and H_2_O_2_ (different concentrations) were dissolved in distilled water at 37°C. No liquid flow was observed within 30 s following tube inversion, which was regarded as the gel point of hydrogel. In this experiment, the concentrations of HRP and H_2_O_2_ used were 5, 10, 25, 50, 75, and 100 U/ml, and 5, 10, 20, 30, 40, and 50 mM, respectively.

### Colony Formation Assay

For colony forming assay, HUVECs were prepared as single cell suspension and seeded in six-well plates (200 cells per well) for 24 h. Then exposed to different treatments of AL (0, 1, 2, 5, 10, and 20 µM), AL-HA-Tyr-5 (0, 1, 2, 5, 10, and 20 µM AL-HA-Tyr + 5 U/ml hyaluronidase) for 24 h separately. Then cells were further incubated at 37°C for 7 days. When the colonies were visible, they were washed twice with phosphate buffered saline, fixed with 4% methanol for 15 min, and stained with 0.05% crystal violet for 30 min. The plates were gently washed twice, and visible colonies were counted under a Leica TE2000-S microscope (Leica Microsystems GmbH, Wetzlar, Germany). (Tokyo, Japan); more than 50 cells were regarded as a colony. Colony inhibition rate (%) was calculated as one-colony counts in sample/colony counts in control (HA-Tyr) × 100%.

### Animals and Tumor Model

The 72 female C57BL/6J (age, 4–6-weeks) mouse xenograft models (Chongqin Tengxing Experimental Animal Center, Chongqin, China) partly overlapped with our previous studies (11). The Institutional Animal Southwest Medical Care and Use Committee (Luzhou, China), approved all the animal experiments in this study, which was implemented in accordance with the guidelines of this committee. The mice were housed in sterile cages without specific pathogens under 12 h/12 h light-dark cycles and provided with standard chow and fresh water daily. The lung cancer model was established by subcutaneously injecting 100 μl of suspended LLC cells (1.5 × 10^7^/ml) into the dorsal side of the right foot of each mouse.

### Treatment

The rate of LLC tumor formation was 100%. The tumor-bearing mice were randomly divided into six groups of 12 each when the tumor volume reached 100–200 mm^3^. The control and AL groups were orally administered with 0.1 ml/day of 0.9% saline and AL, respectively, for 14 days. The other groups received a total of three intratumoral injections (the first, the fifth, the tenth day) of 0.1 ml of HA-Tyr with 50 U/ml HRP (HA-Tyr), or AL-HA-Tyr or RT+AL-HA-Tyr over the 14 days. The AL was orally administered with 3 mg/kg/D (18), and AL-HA-Tyr was intratumorally administered with 14 mg/kg/time. Before the second injection, the mice undergoing RT were fixed in a radiation box. The dorsal side of the right foot of each mouse bearing tumors was covered with oil gauze to protect the skin. The tumor areas were irradiated using an Elekta Precise LINAC delivering 6 mV X-rays at a dose rate of 60 cGy/min, and a radiation dose of 10 Gy. The head and belly of the mice were protected with a 0.5-cm-thick lead shield. Thereafter, six mice in each group were euthanized by cervical dislocation at random, and tumors and internal organs were collected. Tumor hypoxia was detected by micro 18F-FMISO PET/CT imaging. The survival of the remaining six mice was monitored, and then survival curves were created. Tumor growth curves were obtained based on tumor size at 15 days after treatment. Tumor weight and size were measured every 2 days. Tumor volume was calculated using calipers as *(length × width^2^)/*2.

### Micro 18F-FMISO PET/CT

Hypoxia in tumors was evaluated 5 days after treatment by micro 18F-FMISO PET/CT imaging using an Inveon^®^ microPET/CT (Siemens, Munich, Germany). A professional nuclear medicine engineer prepared and purified 18F-FMISO using a fluorine multifunctional label synthesis module. Three mice (weight, 20–24 g) from each group were fasted for 12 h at random, and then 50–100 µCi 18F-FMISO measured and corrected by the radioactivity detector was injected into the caudal veins of the mice. After 1.5–2 h, the mice were anesthetized with 1% pentobarbital sodium (5 mg/kg), and fixed to the central PET ring field using duct tape. The CT images were obtained with the following parameters: 80 kV; current, 500 µA; exposure, 200 ms; total rotations, 220; amplification, ×1.0. Whole body PET images were acquired under the following conditions: acquisition duration at window, 3.4 ns; energy window, 350–650 keV; acquisition duration, 10 min. We reconstructed CT and PET images using the Feldkamp algorithm and 2D-filtered back projection (FBP). The PET/CT images were fused using Inveon^®^ Research Workplace V4.2 software (SIEMENS) and visualized using Maximum intensity projection (MIP).

### Micro 18F-FMISO PET/CT Image Analysis

Sagittal, coronal, transverse, and three-dimensional PET and CT images and fusion images were prepared using the Inveon^®^ Research Workplace V4.2 software. The most obvious position of the tracer indicating the most metabolism was selected as the center of tumor cross sections, and regions of interest (ROIs) were drawn. The maximum standard uptake value (SUV_max_) of the tumor was measured by placing a 3D volume of interest (VOI) around each tumor. The SUVmax of the left thigh muscle was calculated, the muscle background was determined, then the software was applied to draw a 2.5 × 2.5 × 2.5-mm 3D VOI and obtain the SUVmax. The average of three measurements was calculated and taken as the SUVmax of the muscle background. The SUVmax of the tumor was divided by that of the muscle background for normalization to obtain the ratio of the target area (tumor) to the non-target area (muscle) (T/M).

### Histopathology and Immunohistochemistry

The harvested tumors and organs were fixed with formalin, embedded in paraffin, and sliced into 4-μm-thick sections for histopathology and immunohistochemistry. The heart, lung, liver, kidney, and spleen sections were stained with hematoxylin and eosin (HE). The tumor samples were immunostained with antibodies, VEGF-A and HIF-1*α* were stained with DAB, and *γ*-H2AX and Ki-67 were stained with AEC. Images were captured using an optical TE2000-S microscope (Leica). Visible and total numbers of VEGF-A, H2AX, HIF-1*α*, Ki-67 positive cells were counted in six randomly selected regions in each tumor section at 400× magnification, and the ratios (%) of positive cells were calculated. Expression was quantified using Image-ProPlus6.0 software (Media Cybernetics Inc., Bethesda, MD, USA).

### Statistical Analysis

Data are expressed as means ± standard deviation (SD). Results were assessed using unpaired two-tailed t tests. Survival was assessed using Kaplan–Meier curves. All data were analyzed using SPSS statistics 17.0 Software (SPSS Inc, Chicago, IL, USA). Values with P <0.05 were considered statistically significant.

## Results

### AL-HA-Tyr Preparation and Biochemical Characteristics

**Figure 1:**
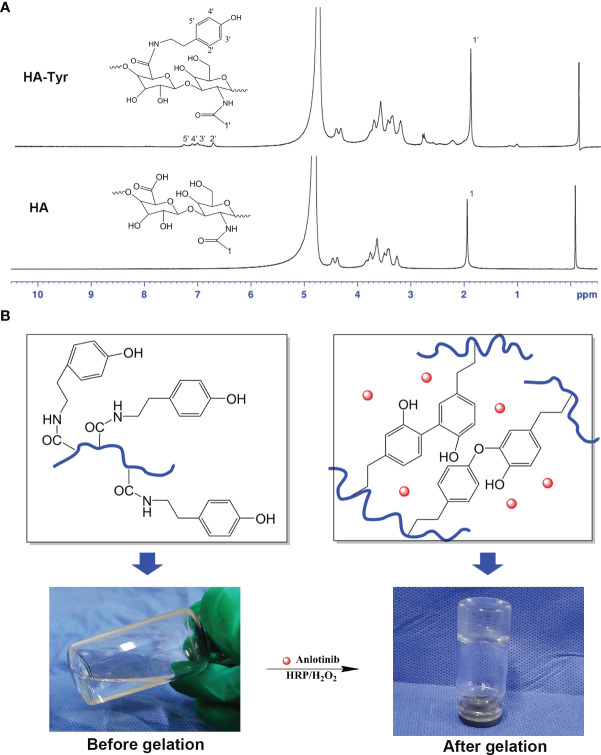
Characteristics of anlotinib hydrogels (AL-HA-Tyr). **(A)** ^1^H-NMR of hyaluronic acid (HA) and hyaluronic acid-tyramine (HA-Tyr). **(B)** Characterization of AL-HA-Tyr hydrogel. Left: HA dissolved in distilled water. Right: AL-HA-Tyr hydrogel formation catalyzed by horseradish peroxidase and H_2_O_2_.

The ^1^H-NMR measurements (**Figure 1A**) showed the proton absorption peaks at *δ* = 6.78 ppm, *δ* = 7.06 ppm, *δ* = 7.17 ppm, and *δ* = 7.32 ppm (2′, 3′, 4′ and 5′ protons) of the tyramine benzene ring. Through *δ* = 6.78 ppm, *δ* = 7.06 ppm, and *δ* = 7.17 ppm, the integral area ratio of proton absorption peak (*δ* = 1.95 ppm) of *δ* = 7.32 ppm, and the HA methyl calculated degree of substitution of tyramine (the number of tyramine groups per 100 HA repeat units) was 18.

Characterization of the AL-HA-Tyr hydrogel is shown in **Figure 1B**. The HA-Tyr (1.0 wt.%) dissolved in distilled water prior to gelation was transparent, colorless, and fluid (left). Mixing it with AL (1 µM) and HRP (5.0 U/ml)/H_2_O_2_ (20 mM) following gelation caused it to have in a colorless, transparent, and non-fluid semi-solid nature (right). AL-HA-Tyr hydrogel was formed following oxidative coupling of tyramine moieties catalyzed by H_2_O_2_ and HRP (**Figure 1B**).

**Figure 2:**
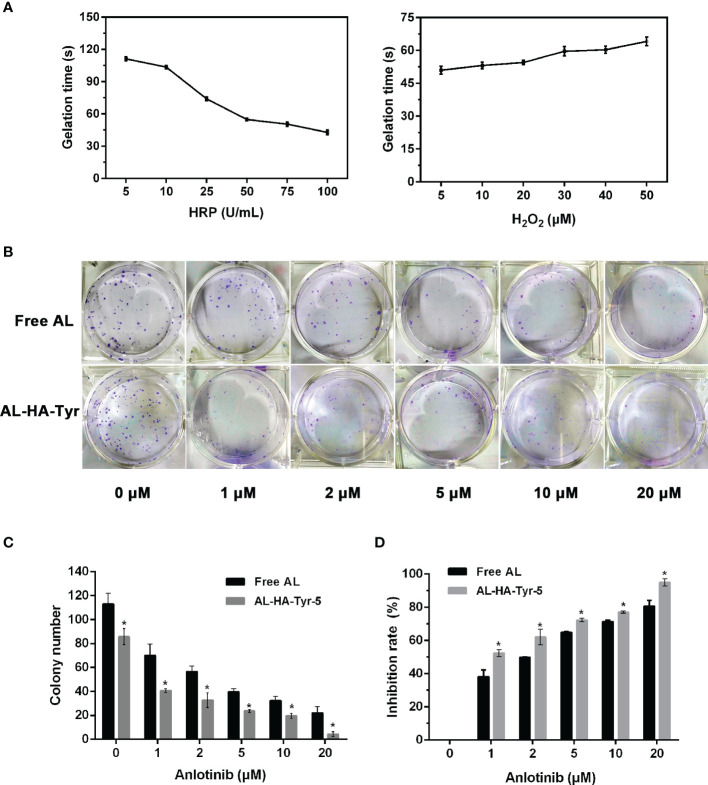
Factors affecting gel formation and effects of anlotinib hydrogel (AL-HA-Tyr) on colony formation. **(A)** Gelation points of hyaluronic acid-tyramine (HA-Tyr) conjugates at different concentrations of H_2_O_2_ and horseradish peroxidase (n = 6). Data are shown as means ± SD. **(B)** Representative images of colony formation assays using human umbilical endothelial cells (HUVECs) incubated with various concentrations of anlotinib (AL) or anlotinib hydrogels (AL-HA-Tyr) + lysozyme-5. **(C)** Quantitation of colony formation of HUVEC *in vitro*. **(D)** Inhibition rate of colony formation in AL and AL-HA-Tyr-5. Data are shown as means ± SD. (n = 3). *P < 0.05, (Free AL *vs.* AL-HA-Tyr-5).

The phase transition was determined by the test tube inversion method. As depicted in **Figure 2A**, maintaining a constant concentration of H_2_O_2_, while increasing the concentration of HRP from 5 U/ml to 100 U/ml caused the gelation time of HA-Tyr hydrogel to decrease from ~110 s to ~40 s, while the gelation rate increased with the increase of HRP concentration. When the concentration of HRP was kept constant while changing the concentration of H_2_O_2_, the gelation time changed from ~50 s to ~70 s, therefore the gelation rate of hydrogel can be more controlled by changing the concentration of HRP in the catalytic system of HRP/H_2_O_2_.

### Inhibitory Effect of AL-HA-Tyr

The ability of HUVECs incubated with AL-HA-Tyr-5 to form colonies was evaluated (**Figure 2B**). Increasing the concentration of AL or AL-HA-Tyr-5 significantly and dose-dependently inhibited colony formation. The number of colonies formed when incubated with 0 and 20 μM AL and with 0 and 20 μM AL-HA-Tyr was 113 and 22 *vs.* 85 and 4, respectively (P < 0.05) (**Figure 2C**). The rates of colony inhibition by 1 and 20 μM AL were 38.2 and 80.7%, respectively, *vs.* 52.4 and 95% by AL-HA-Tyr-5 (P < 0.05) (**Figure 2D**). Overall, AL-HA-Tyr-5 inhibited HUVEC colony formation more effectively than AL at all concentrations (P < 0.05).

### RT + AL-HA-Tyr Enhanced Anti-Tumor Effects

**Figure 3:**
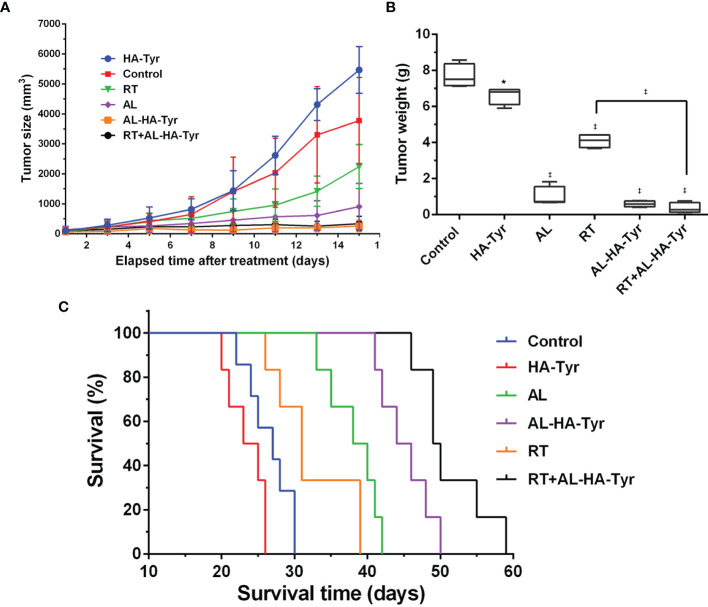
Tumor growth was inhibited, and survival was prolonged by radiotherapy (RT) + anlotinib hydrogel (AL-HA-Tyr) in LLC model. **(A)** Tumor size at indicated times (n = 6). **(B)** Weight of excised tumors. (n = 4; *P < 0.05, and ^‡^P < 0.001,Control *vs.* hyaluronic acid-tyramine (HA-Tyr), anlotinib (AL), AL-HA-Tyr, RT, and RT + AL-HA-Tyr P < 0.01, RT *vs.* AL-HA-Tyr+ RT. **(C)** Survival of mice in each group (n = 6).

The treatment was terminated after 14 days, and the tumor volume was measured every two days (**Figure 3A**). The order of tumor growth rate was HA, control, RT, AL, AL-HA-Tyr, and RT + AL-HA-Tyr. The tumor weight of control was the heaviest, followed by HA-Tyr, RT, AL, AL-HA-Tyr, and RT + AL-HA-Tyr (**Figure 3B**). The tumor weight of control was 13 times that of AL-HA-Tyr (7.7 ± 0.3 *vs.* 0.6 ± 0.1), indicating that AL-HA-Tyr had an inhibitory effect on tumor growth (p < 0.001). The tumor weight of RT was 11 times that of RT + AL-HA-Tyr (4.1 ± 0.2 *vs.* 0.4 ± 0.1), indicating that RT + AL-HA-Tyr had obvious inhibitory effect on tumor growth (P < 0.001).

The HA-Tyr, control, RT, AL, AL-HA-Tyr, and RT + AL-HA-Tyr groups survived for 26, 30, 39, 42, 50, and 59 days respectively (**Figure 3C**). RT + AL-HA-Tyr significantly prolonged mouse survival by 29 days compared with the control (P < 0.001). Overall, RT + AL-HA-Tyr prolonged survival more than any other treatment (all P < 0.001).

### AL-HA-Tyr Reduced Toxic and Side Effects

**Figure 4:**
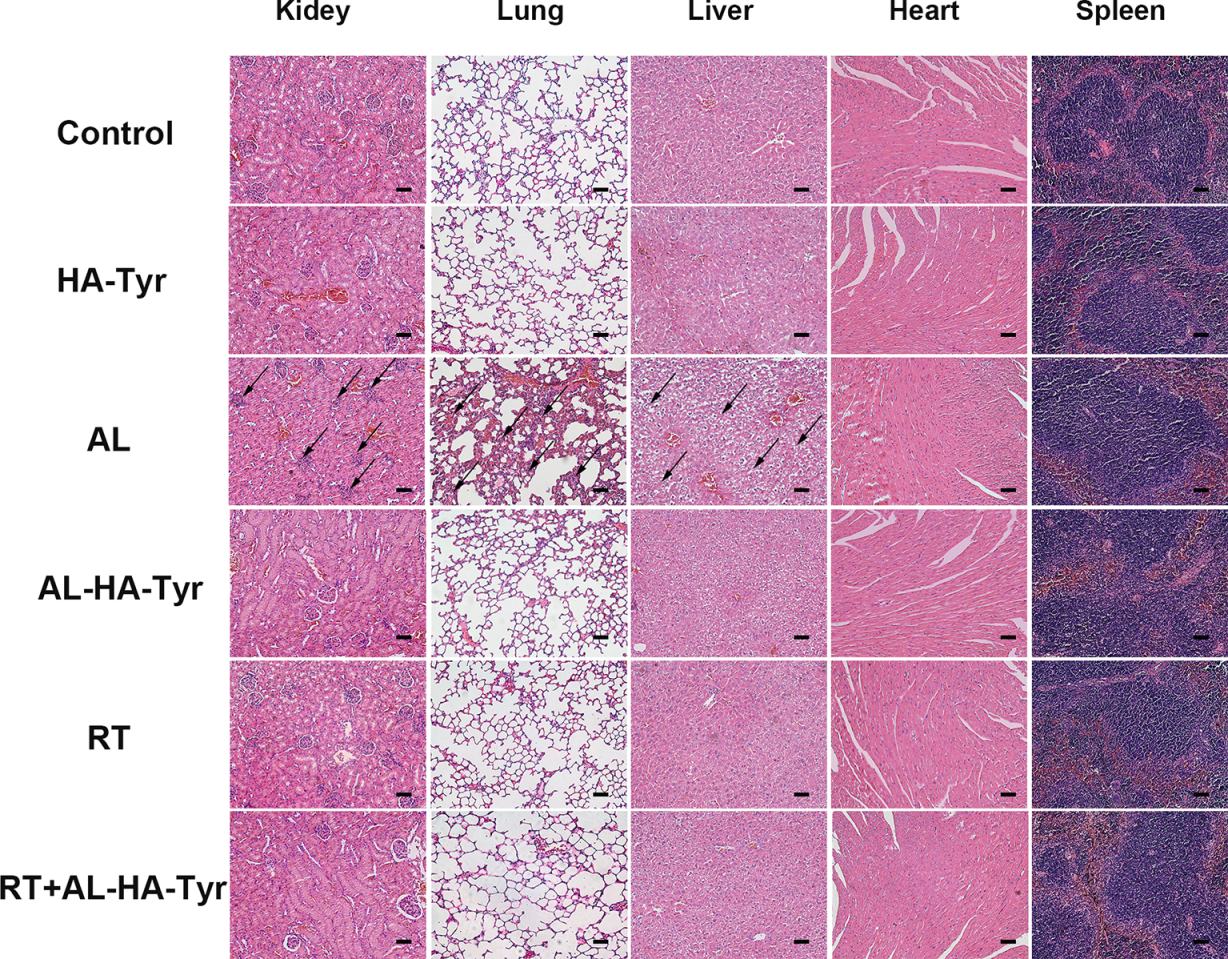
Histopathology of organs in Lewis lung carcinoma (LLC) tumor-bearing mice. Toxicity evaluated by hematoxylin and eosin (HE) staining visceral tissues. Scale bar = 50 μm.

Toxicity was evaluated using visceral tissue H&E staining in this assay (**Figure 4**). The heart and spleen were the same in each group, while the kidney, lung, and liver differed. The morphology of visceral tissue is normal in control, HA-Tyr, RT, AL-HA-Tyr, and RT+AL-HA-Tyr. However, AL showed various renal corpuscles atrophy in kidney; alveolar cells proliferated obviously in lung; hepatocytes showed balloon-like degeneration in liver. Indeed, AL-HA-Tyr reduced the visceral toxicity of mice.

### RT+AL-HA-Tyr Decreased 18F-FMISO Uptake

**Figure 5:**
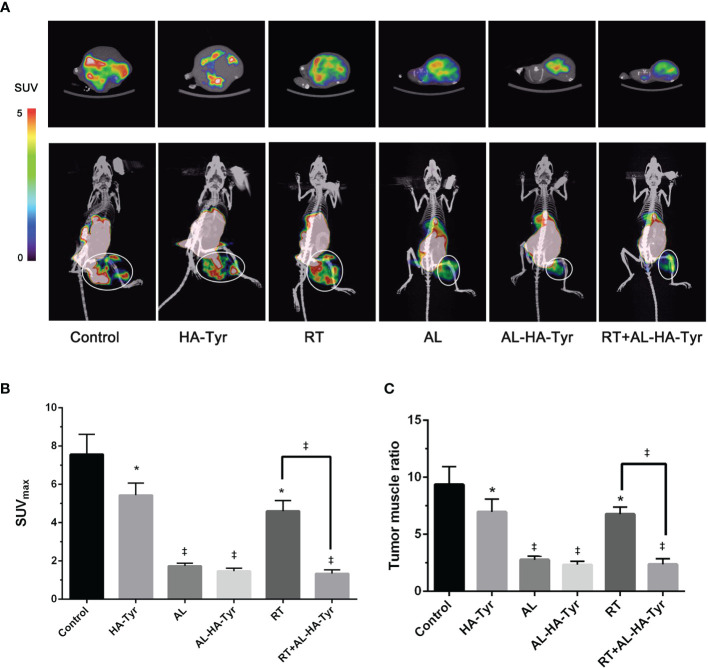
Hypoxic image of 18F-FMISO PET/CT after treatment, maximum standard uptake value (SUVmax) of ROI, and tumor muscle ratio (T/M) in each group. **(A)** Representative 18F-FMISO PET/CT MIP fusion image (rainbow color scale). The upper image is a cross section of the tumor; the lower image is the coronal surface of the tumor. The ellipses in the images are the regions of interest (ROIs) in the tumor target. **(B)** SUVmax of ROI in each group. **(C)** T/M in each group. (n = 3) *P < 0.05, and ^‡^P < 0.001, control *vs.* hyaluronic acid-tyramine (HA-Tyr), anlotinib (AL), anlotinib hydrogel (AL-HA-Tyr), radiotherapy (RT), and AL-HA-Tyr+ RT, respectively; RT *vs.* AL-HA-Tyr+ RT.

**Figure 5** shows representative 18F-FMISO PET/CT MIP images and the SUV_max_ and T/M of tumor-bearing mice. The uptake of 18F-FMISO in the combined group was decreased and hypoxia was significantly improved in the RT+AL-HA-Tyr, compared with the RT group (1.3 ± 0.1 *vs.* 4.6 ± 0.3 p < 0.001). The AL and AL-HA-Tyr groups did not significantly differ, indicating similar effects on tumor hypoxia (1.7 ± 0.1 *vs.* 1.5 ± 0.1, p > 0.05). The T/M results (**Figure 5C**) were the same as those for the tumor SUV_max_. Uptake of 18F-FMISO was high in the control and HA groups (9.4 ± 0.9 and 7.0 ± 0.6, respectively), but significantly reduced in the AL and AL-HA-Tyr groups compared with the control (2.8 ± 0.2 and 2.3 ± 0.2 *vs.* 9.4 ± 0.9, p < 0.001). The T/M was significantly decreased in the RT+AL-HA-Tyr compared with the RT group (2.4 ± 0.3 *vs.* 6.8 ± 0.4, p < 0.001).

### RT + AL-HA-Tyr Decreased Ki67 and Increased *γ*-H2AX levels

**Figure 6:**
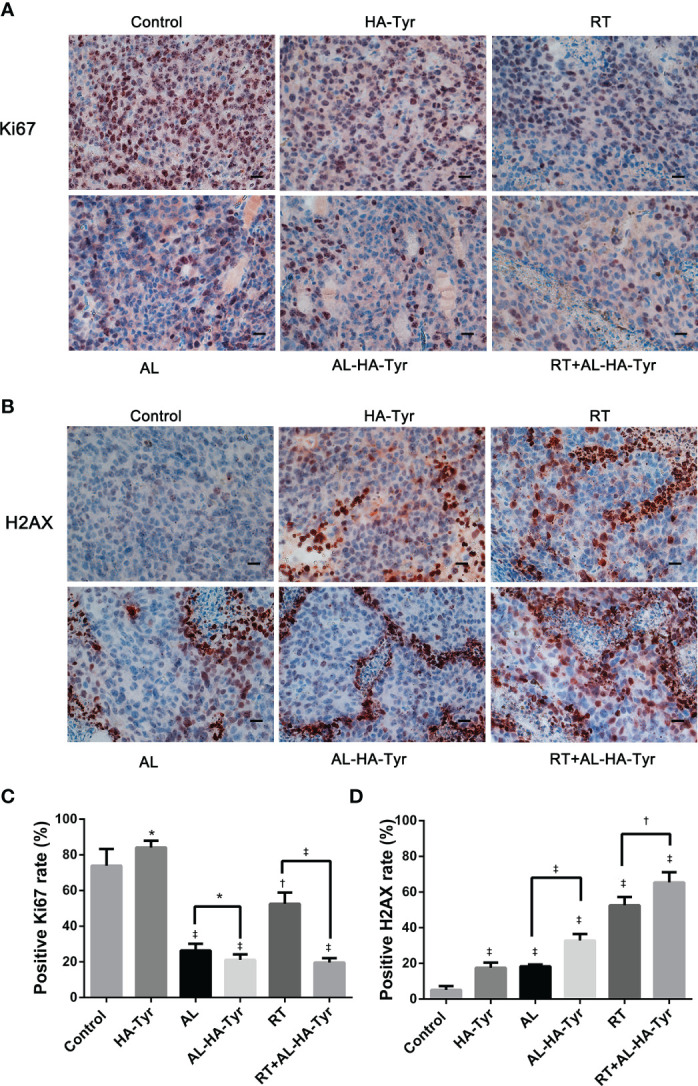
Ki-67 and *γ*-H2AX expression in transplanted tumors from different groups. **(A**, **B)** Representative immunohistochromatography images showing Ki-67 and *γ*-H2AX expression in tumor tissues. Scale bar = 20 μm. **(C**, **D)** Histogram showing percentage of Ki-67 and *γ*-H2AX positive cells in each group. Data are expressed as mean ± SD. *P < 0.05, ^†^P < 0.01, ^‡^P < 0.001; control *vs.* hyaluronic acid-tyramine (HA-Tyr), anlotinib (AL), anlotinib hydrogel (AL-HA-Tyr), radiotherapy (RT), RT+AL-HA-Tyr; AL *vs.* AL-HA-Tyr, RT *vs.* RT+AL-HA-Tyr.

Proliferation and cell damage were evaluated as Ki-67 and *γ*-H2AX values (**Figure 6**) in tumor tissues. The relative ratios of Ki-67 positive cells were: RT+AL-HA-Tyr (19.8 ± 1.0%), AL-HA-Tyr (21.3 ± 1.0%), AL (27.0 ± 1.1%), RT (53.3 ± 2.2%), control (75.8 ± 3.0%), and HA-Tyr (84.8 ± 1.4%). The Ki-67 value was higher in AL than in AL-HA-Tyr (P < 0.05), and in RT+AL-HA-Tyr than in AL-HA-Tyr (P < 0.001; **Figure 6C**). Significantly more cells were positive for γ-H2AX in tumor tissues of the RT + AL-HA-Tyr, than the RT and control groups (65.3 ± 2.4% *vs.* 52.5 ± 1.9% and 5.2 ± 0.9%; p < 0.01, p < 0.001, respectively; **Figure 6D**).

### RT + AL-HA-Tyr Decreased VEGF-A and HIF-1*α*

**Figure 7:**
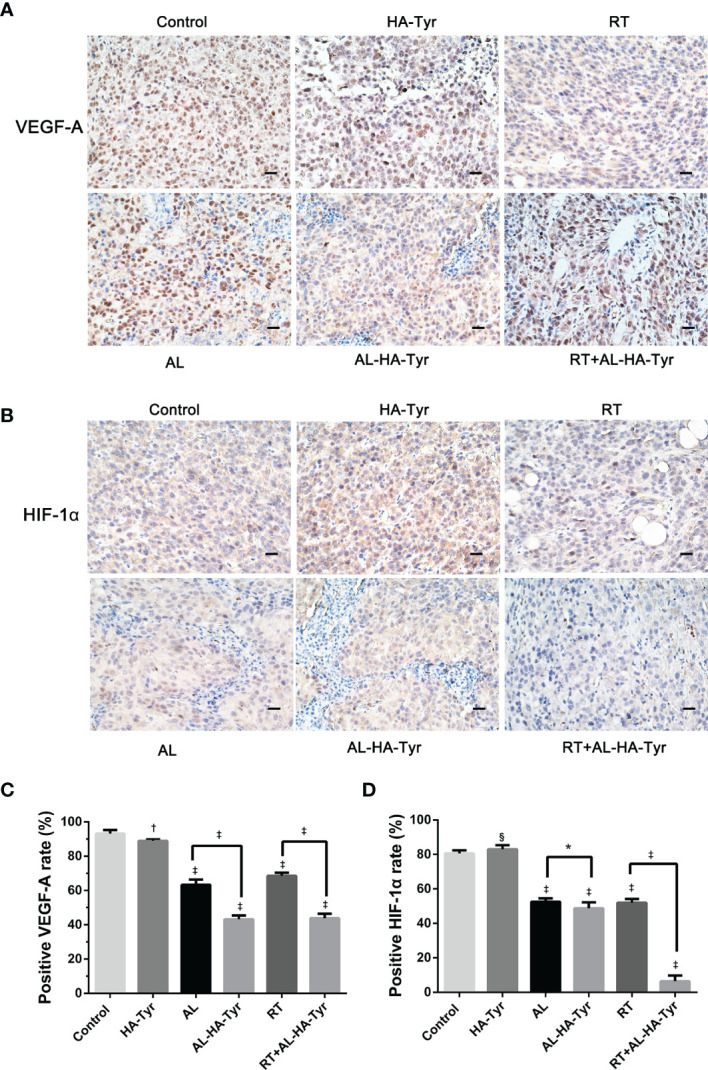
HIF-1*α* and VEGF-A expression in transplanted tumors from different groups. **(A**, **B)** Representative immunohistochromatography images showing HIF-1*α* and VEGF-A expression in tumor tissues. Scale bar = 20 μm. **(C**, **D)** Histogram showing percentage of HIF-1*α* and VEGF-A positive cells in each group. Data are expressed as means ± SD. *P < 0.05, ^†^P < 0.01, ^‡^P < 0.001, ^§^P > 0.05: Control *vs.* hyaluronic acid-tyramine (HA-Tyr), anlotinib (AL), anlotinib hydrogel (AL-HA-Tyr), radiotherapy (RT), RT+AL-HA-Tyr; AL *vs.* AL-HA-Tyr, RT *vs.* RT+AL-HA-Tyr.

**Figure 7** shows that RT + AL-HA-Tyr decreased VEGF-A and HIF-1*α* values. The relative ratios of VEGF-A positive cells were as follows: RT+AL-HA-Tyr (43.8 ± 1.1%), AL-HA-Tyr (68.5 ± 0.8%), AL (63.3 ± 1.3%), RT (68.5 ± 0.8%), HA-Tyr (88.8 ± 0.5%), and control (93.2 ± 0.9%). The VEGF-A ratio was higher in AL than in AL-HA-Tyr, and in RT+AL-HA-Tyr than in AL-HA-Tyr (P < 0.001 for both; **Figure 7C**). Significantly fewer HIF-1*α* cells were positive in tumor tissues of the RT + AL-HA-Tyr, than the RT group (6.3 ± 1.4% *vs.* 52.0 ± 0.8%, P < 0.001; **Figure 7D**), indicating that RT + AL-HA-Tyr significantly improved hypoxia. Ratios of HIF-1*α* positive cells were similar in the AL-HA-Tyr AL and RT groups (48.7 ± 1.4%, 52.5 ± 0.8%, and 52.0 ± 0.8%, respectively, p > 0.05) indicating similar degrees of tumor hypoxia.

## Discussion

Tumor blood vessels offer an important target for cancer management (19), which is associated with the degree of tumor hypoxia and anti-tumor effects. Hypoxic microenvironments as associated with more aggressive and invasive tumor cells (13, 20–23). Anlotinib hydrochloride is a new and promising antiangiogenesis agent with broad anti-tumor capacity (24–28). AL exerts anti-vascular and anti-tumor effects, and tumor shrinkage is beneficial to RT. RT-induced resistance and hypoxia can be improved through the ability of AL to downregulate the angiogenesis. RT combined with AL in tumor locales might achieve 1+1 > 2 effects. Therefore, we combined AL with a hyaluronic acid hydrogel for effective and practical local drug delivery. We encapsulated AL with HA-Tyr hydrogel (AL-HA-Tyr) and confirmed its synthesized properties using proton nuclear magnetic resonance (^1^H-NMR).

The inhibitory effect of the AL-HA-Tyr hydrogel on HUVEC was verified *in vitro*. AL-HA-Tyr-5 enhanced the inhibition of HUVEC colony formation and reduced the numbers of colonies compared with free AL in a dose-dependent manner, consistent with previous studies (29–32). Its antiangiogenesis effect was evaluated by detecting the expression of VEGF-A in LLC tumor-bearing mice. We found that, compared with AL, AL-HA-Tyr decreased the expression of VEGF-A; compared with RT, RT+AL-HA-Tyr combined therapy could significantly reduce the proportion of VEGF-A positive cells. AL-HA-Tyr showed significant anti-angiogenic effect.

*In vivo*, AL-HA-Tyr’s safety was also evaluated. The uptake of AL in solid tumors was extremely low whereas it was higher in visceral organs (33). Therefore, we selected animal visceral HE staining to examine the toxic effect of AL on viscera. We observed that AL was highly toxic to the lung, liver, and kidney of mice, while AL-HA-Tyr had no obvious visceral toxicity to C57BL/6J mice. The same AL doses were administered orally and intratumorally when injected as AL-HA-Tyr. Changing the drug delivery route from oral to direct intratumoral injection decreased the frequency of administration and alleviated toxicity. This might have been due to the controlled-release effect of AL-HA-Tyr that resulted in the delivery of an effective dose into the tumor while decreasing blood AL concentrations, which in turn diminished systemic toxicity. Regardless, the effectiveness and safety of AL-HA-Tyr was verified.

Based on the tumor weight, tumor volume and survival, the anti-tumor effects of AL-HA-Tyr and AL, were similar, but AL-HA-Tyr significantly prolonged survival, mainly because of sustained release of AL from the hydrogel and inhibitory effects at tumor sites, and delayed tumor growth. Furthermore, the direct delivery of AL-HA-Tyr into tumors increased the effects while reducing the visceral toxicity of AL. Compared with RT, RT+AL-HA-Tyr significantly inhibited tumor growth and prolonged the survival of tumor-bearing mice. We also showed that RT + AL-HA-Tyr significantly increased the expression of γ-H2AX and decreased that of Ki67, indicating positive anti-tumor effects. These results might be attributed to the potentially improved oxygen delivery and increased efficiency of RT conferred by antiangiogenesis agents. In short, RT+AL-HA-Tyr enhanced anti-tumor effects and prolonged the survival of mice harboring LLC tumors.

Targeted molecular imaging of hypoxia offers an attractive, minimally invasive, and repeatable assessment of hypoxia and its reversibility (34). Functional PET/CT hypoxia imaging can directly display areas and degrees of tumor hypoxia (35–38). We used micro 18F-FMISO PET/CT to evaluate hypoxia in mice treated with AL and radiation (**Figure 5**). The radioactivity of 18F-FMISO is mainly distributed in the abdominal organs such as intestines, kidneys, and liver of healthy mice. This explains the apparently excessive FMISO uptake in the abdominal regions of our mice (**Figure 5**). It is used for PET imaging malignant lung tumors, as it can clearly distinguish tumor from normal tissues and thus has important diagnostic value. The 18F-FMISO uptake of RT+AL-HA-Tyr was significantly lower than that of RT, showing that the combination with AL-HA-Tyr improved hypoxia after RT. Using the T/M ratio for normalization yielded results that were consistent with those of tumor SUVmax. The SUVmax or T/M of 18F-FMISO in PET/CT hypoxia images were essentially the same for AL-HA-Tyr, AL, and RT+AL-HA-Tyr. This might have been due to the similar anti-tumor effects on tumor size. However, regulation of the response to hypoxia differed. The effects of hypoxia are controlled by the activity of the transcription factor, hypoxia-inducible factor-1 (HIF-1), which plays an important role in cellular responses to hypoxia (39, 40). Our immunohistochemical assessment of HIF-1α expression in tumors showed that AL-HA-Tyr reduced HIF-1*α* expression compared with AL and that RT+AL-HA-Tyr significantly reduced it compared with any other group. The difference between 18F-FMISO uptake and HIF-1*α* expression might be associated with a delay in expression. Overall, RT+AL-HA-Tyr improved tumor hypoxia cellular responses more than any other treatment assessed herein.

Here, we synthesized AL-HA-Tyr, verified its inhibitory effect on HUVEC, and determined its effectiveness and safety in LLC tumor-bearing mice. When combined with RT, AL-HA-Tyr enhanced anti-tumor effects, prolonged survival, and improved tumor hypoxia to a greater extent than any of the other therapies, but the molecular mechanism remains to be elucidated. We used only one kind of lung cancer cell for the study, and whether other tumor cells behave in this way warrant further investigations. Since AL has broad anti-tumor effect in solid tumor, the same results may be found in other type of tumors. We predict that AL-HA-Tyr combined with RT will have a significant effect on the treatment and prognosis of patients and hypoxia combined with imaging observation can be used in the follow-up evaluation of solid cancer during treatment in the future.

## Conclusions

We synthesized AL-HA-Tyr and validated its safety and efficacy in a mouse model of LLC. This combination dose-dependently inhibited the growth and colony formation of HUVECs. The anti-tumor effects and degree of improvement in hypoxia were similar between AL-HA-Tyr and AL, but AL-HA-Tyr reduced visceral toxicity and prolonged survival. RT+AL-HA-Tyr more significantly reduced 18F-FMISO uptake as well as HIF-1*α*, Ki67, and VEGF-A expression, and increased *γ*-H2AX expression in tumor cells compared to the other treatments. Taken together, RT+AL-HA-Tyr improved tumor hypoxia, enhanced anti-tumor effects, and prolonged the survival of LLC-bearing mice.

## Data Availability Statement

The original contributions presented in the study are included in the article/supplementary material. Further inquiries can be directed to the corresponding authors.

## Ethics Statement

The animal study was reviewed and approved by the Institutional Animal Southwest Medical Care and Use Committee (Luzhou, China).

## Author Contributions

QG: conceptualization, methodology, resources, software, data curation, methodology, formal analysis, investigation, visualization, writing—original draft, and writing—review & editing. YJ: data curation, methodology, investigation, validation, and formal analysis. XL: data curation, validation, and resources. HuC: investigation and validation. ST: visualization and investigation. HaC: investigation and data curation. XS: visualization and investigation. YC: visualization, investigation, supervision, and funding acquisition. SF: supervision, validation, resources, formal analysis, and writing—review & editing. SL: conceptualization, funding acquisition, supervision, project administration, and writing—review & editing. All authors contributed to the article and approved the submitted version.

## Funding

This work was supported by grants from the Scientific Research Foundation of the Luzhou Science and Technology Bureau (No. 2016LZXNYD-J05), Key Laboratory of Nuclear Medicine and Molecular Imaging of Sichuan Province (No. HYX19008).

## Conflict of Interest

The authors declare that the research was conducted in the absence of any commercial or financial relationships that could be construed as a potential conflict of interest.
